# Factors Related to Metabolic Syndrome Development and Recovery in Chinese Adults: A Prospective Longitudinal Study

**DOI:** 10.3389/fendo.2022.923650

**Published:** 2022-06-13

**Authors:** Chenyu Zhang, Sisi Fang, Haoyu Wang, Zhongyan Shan, Yaxin Lai

**Affiliations:** ^1^ Department of Endocrinology and Metabolism, The National Health Committee (NHC) Key Laboratory of Diagnosis and Treatment of Thyroid Diseases, Institute of Endocrinology, The First Hospital of China Medical University, Shenyang, China; ^2^ Fuxin Central Hospital, Fuxin, China

**Keywords:** metabolic syndrome, hyperuricemia, visceral obesity, BMI, HDL-C (high density lipoprotein-cholesterol)

## Abstract

**Objective:**

This study was a prospective assessment of the epidemiological characteristics of metabolic syndrome (MetS) in cities in Northeast China. We explored the factors that affect the occurrence and outcome of MetS according to sex.

**Design and Methods:**

This was a longitudinal survey assessing MetS status among 750 urban community residents in China. At baseline, the intra-abdominal fat area was measured by MRI, simple anthropometric parameters (body mass index (BMI), waist circumference (WC), etc.) were used to evaluate fat distribution; blood pressure and blood lipid profile were measured; an oral glucose tolerance test (OGTT) was used to detect blood glucose; questionnaires were used to investigate lifestyles. Follow-up was conducted after 1.5 years (follow-up rate was 66.93%) to analyze the incidence of MetS and the influencing factors of MetS outcomes according to sex.

**Results:**

The 1.5-year cumulative incidence of MetS in the survey area was 25.40%. Men with visceral obesity were more likely to develop MetS than those with subcutaneous obesity (OR=9.778, p<0.05). Increased BMI (OR=1.379) and blood uric acid (BUA)>416 mmol/L (OR=2.318) were associated with the occurrence of MetS in men (all p<0.05). At the initial visit, BUA>356.9 mmol/L (OR=3.538), increased BMI (OR=1.212), and increased HbA1c (OR=2.577) were associated with the occurrence of MetS in women (all p<0.05). After 1.5 years, 25.37% of MetS patients no longer had MetS. Elevated diastolic blood pressure (DBP) (OR=1.097) and increased visceral fat (OR=1.023) at the initial visit made men with MetS less likely to recover from MetS (all p<0.05). Higher High-density lipoprotein cholesterol (HDL-C) at the initial visit made women with MetS more likely to recover from MetS (β: -3.509, OR=0.003, p<0.05).

**Conclusion:**

There are different risk factors for MetS in different genders. Hyperuricemia is a risk factor for the onset of MetS in both men and women.

## Introduction

Metabolic syndrome (MetS) is a group of metabolic disorders including obesity, elevated blood glucose, dyslipidemia and hypertension (1). Globally, approximately 25% of adults suffer from MetS, and this proportion is expected to continue to increase in the coming decades (2). MetS is a risk factor for cardiovascular and cerebrovascular diseases and diabetes and a substantial challenge to public health (3, 4).

There are many risk factors for the development of MetS (5). Among them, obesity, particularly central obesity, is a key risk factor and has become one of the core diagnostic indicators for MetS. However, waist circumference (WC) alone cannot adequately identify central obesity caused by increased subcutaneous fat and increased visceral fat. Studies have indicated that visceral fat is more likely to lead to insulin resistance than subcutaneous fat in the abdomen (6). Computed tomography (CT) and magnetic resonance imaging (MRI) are gold standards for differentiation. The roles of visceral and subcutaneous fat in the development and outcome of MetS still need to be further explored.

Both hyperuricemia and MetS are common chronic diseases, and studies have indicated that hyperuricemia and MetS are closely related (7–9). A cross-sectional study indicated that hyperuricemia was an important factor in MetS, but it was not discussed individually in men and women (8). A cross-sectional study from southwestern China demonstrated significant risk factors for MetS with hyperuricemia in people over 80 years of age (7). Nonetheless, cross-sectional studies cannot analyze cause and effect very well. A prospective study with up to 4.5 years of follow-up explored the relationship between new-onset MetS and hyperuricemia and suggested a bidirectional effect (9). However, the role of hyperuricemia in the recovery of MetS is unclear.

Increasing age is also a risk factor for MetS. Studies in the United States have shown that the prevalence of MetS increases significantly after 30 years of age (10). Sex also plays a key role in the development and outcome of MetS due to the presence of sex and sex-related determinants of each single component of MetS. A large Korean cross-sectional study showed that male MetS patients had significantly higher rates of MetS components such as hypertriglyceridemia (+5%), hypertension (+6%), and hyperglycemia (+11%) than female patients, whereas the incidence of central obesity (+12%) and low-density lipoproteinemia (+47%) was significantly higher in female MetS patients than in males (11). In addition, the role of sex in MetS is also age-related. In a cross-sectional study of 4,289 subjects, the overall prevalence of MetS was higher in men than in women (22.3% in men and 15.8% in women), but this association reversed after age 60 years (30.4% in men and 40.3% in women) (12). Evidence suggests that changes in women’s hormonal status during and after menopause affect the development of MetS (13). In addition, many studies have shown that sex may influence cardiovascular risk associated with MetS and response to therapeutic interventions and therefore requires our attention (14).

Studies have shown that MetS can be improved or remission. For example, lifestyle intervention and exercise can have a positive effect on the outcome of MetS and reduce the severity of its components (15, 16). In addition to dietary control and regular exercise, current treatment includes the treatment of antihypertensive, hypoglycemic, regulating dyslipidemia. Exploring which initial conditions are associated with better or worse MetS outcomes will help guide us to choose individualized treatment strategies based on patient conditions.

Because MetS is associated with many diseases, many prospective studies have used MetS as a risk factor to explore the occurrence, development and outcome of related diseases, but few studies have conducted prospective investigations on the development and outcome of MetS. This prospective longitudinal study investigated the prevalence of MetS in Chinese individuals and the risk factors for MetS according to sex and conducted a 1.5-year follow-up to explore the outcomes of patients with MetS to provide theoretical support for clinical practice.

## Materials and Methods

### Study Population

**Figure 1:**
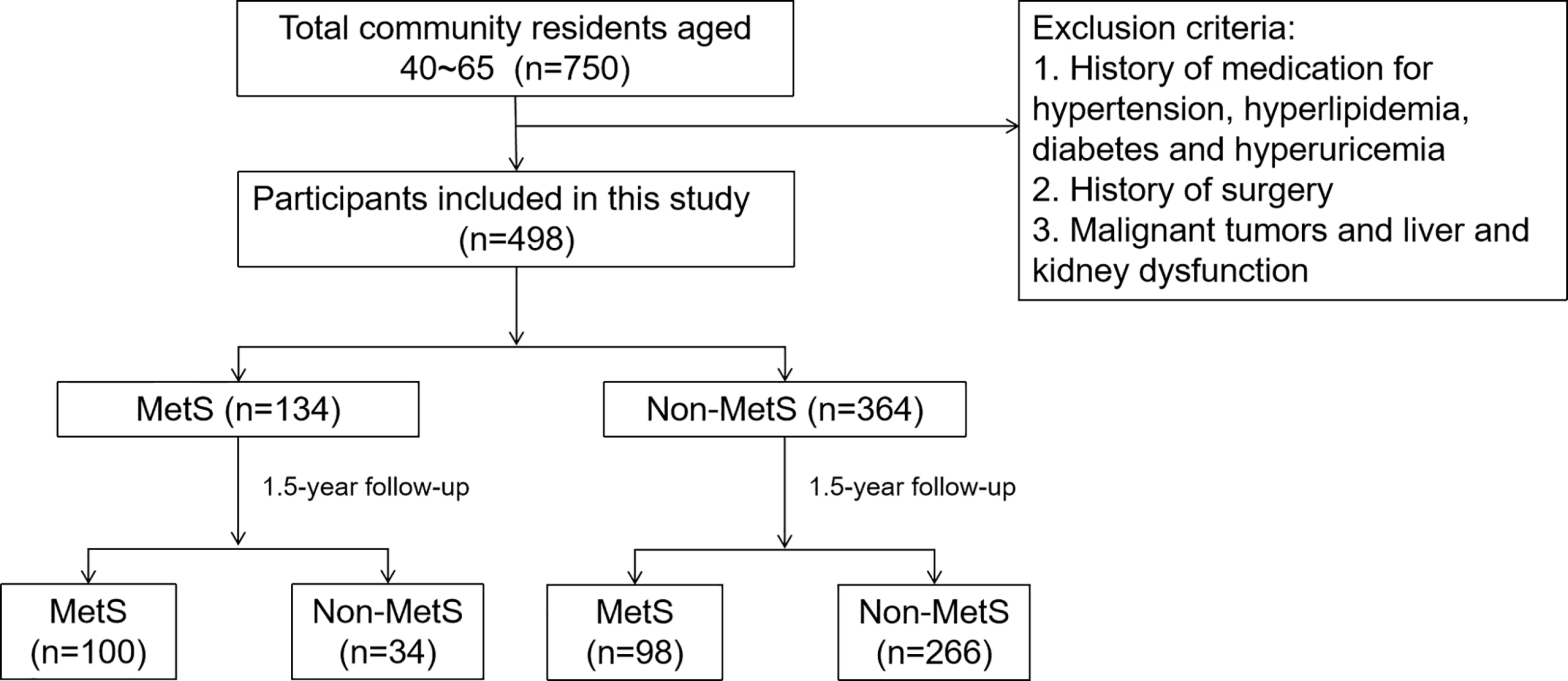
Study flow chart. MetS, metabolic syndrome.

To assess the prevalence and outcome of MetS in the Chinese population, we conducted a community-based prospective study in Shenyang, Liaoning Province, China, in 2011. The study recruited 40- to 65-year-old residents of the area. A total of 750 subjects were included in this epidemiological survey and were followed up for 1.5 years. The exclusion criteria were (1) subjects with a history of medication for hypertension, hyperlipidemia, diabetes or hyperuricemia, (2) subjects with a history of surgery, and (3) subjects with malignant tumors or liver or kidney dysfunction during the baseline period. Ultimately, 498 participants were included. Among them, there were 134 patients with MetS and 364 patients with non-MetS (**Figure 1**).

### Data Collection

A unified questionnaire was used to conduct health surveys for all study subjects at both baseline and the end of follow-up. The main contents of the questionnaire included general information (name, race, occupation, marital status, birth weight, etc.), personal behavior and lifestyle (alcohol consumption, physical activity, eating habits, etc.), family history of diseases (hypertension, diabetes, blood lipids, abnormality, stroke, coronary heart disease, obesity, etc.); and history of illness (diabetes, hypertension, dyslipidemia, etc.). A physical examination was performed to measure height, weight, waist circumference, hip circumference, blood pressure, and heart rate. The percentage of fat content was determined with a body fat meter. All subjects were in the prone position when they underwent an MRI scan of the abdomen between the 4th and 5th lumbar vertebrae at baseline and at the endpoint (field angle (FOV) 42 cm×42 cm, thickness 1 cm, six layers, GE, USA). Two experienced researchers used SLICE-O-MATIC version 4.2 software (Tomovision) to calculate the subcutaneous fat and visceral fat contents. Participants underwent a standard 75 g oral glucose tolerance test (OGTT). Venous blood samples were drawn, and fasting plasma glucose (FPG), 0.5-hour plasma glucose (0.5-h PG) and 2-hour plasma glucose (2-h PG) were measured using the hexokinase method. Fasting nonanticoagulated venous blood samples were taken to determine HbA1c, total cholesterol (TC), triglycerides (TGs), low-density lipoprotein cholesterol (LDL-C), high-density lipoprotein cholesterol (HDL-C) and blood uric acid (BUA) by enzymatic colorimetry.

### Diagnostic Criteria

The 2005 IDF diagnostic criteria are used for the diagnosis of MetS in our study (17): prerequisites: (1) Central obesity: WC ≥90 cm in men; ≥80 cm in women; at least two of the following conditions: (2) SBP ≥130 mmHg, DBP ≥85 mmHg or antihypertensive treatment; (3) TGs ≥ 1.7 mmol/L and/or corresponding lipid-lowering treatment; (4) HDL-C < 1.03 mmol/L in men or <1.29 mmol/L in women and/or corresponding lipid-lowering therapy; and (5) FPG ≥ 5.6 mmol/L or diagnosis and treatment of diabetes. Patients treated with antihypertensive drugs, lipid-lowering drugs and hypoglycemic drugs were not included in this study.

Diagnosis of obesity type: Necessary conditions (18): Body mass index (BMI) ≥ 25.0 kg/m^2^ and waist circumference (WC) ≥ 85 cm for men or ≥ 80 cm for women. Visceral obesity: (1) Male: visceral fat area (VFA) ≥80 cm^2^; subcutaneous obesity: VFA <80 cm^2^; (2) Female: age ≥50 years, VFA ≥90 cm^2^; subcutaneous obesity: VFA <90 cm^2^; age <50 years old, VFA ≥70 cm^2^; subcutaneous obesity: VFA <70 cm^2^.

### Statistical Analyses

All data were analyzed by SPSS 23.0 software. We used the Kolmogorov–Smirnov test for normality testing. Data are expressed as the mean ± standard deviation (SD), median (IQR) and count (percentage). The t-test was used for the mean comparison of variables that conformed to a normal distribution, and the rank-sum test was used for the variables that conformed to a skewed distribution. The chi-square test was used to compare the incidence and other rates of MetS. Univariate logistic analysis (enter) was used for selecting independent variables. Multivariate logistic regression (enter) was used for influencing factor analysis. P<0.05 indicated that a difference was statistically significant.

## Results

### Baseline Characteristics of the Participants

A total of 750 people were included in this study in 2011, and a total of 502 people were followed up at 1.5 years, with a follow-up rate of 66.93%. There was no significant difference in the follow-up rate according to sex (p>0.05). A total of 498 people (269 men and 229 women) were included in this study. Among them, there were 134 patients with MetS and 364 without MetS. At baseline, 94 men (18.87%) and 40 women (8.03%) were diagnosed with MetS (**Table 1**).

**Table 1:** General Characteristics.

	MetS	Non-MetS	p
Participants	134	364	–
Age	52.9 ± 6.7	53.6 ± 7.8	0.017
Male/Female	94/40	175/189	0.000
**Alcohol consumption, N (%)**			0.021
Never	63 (47.0%)	220 (60.4%)	
Occasional	36 (26.9%)	66 (18.1%)	
Frequent	35 (26.1%)	78 (21.4%)	
**Diet, N (%)**			0.178
Meat	16 (11.9%)	25 (6.9%)	
Meat/Vegetables	60 (44.8%)	166 (45.6%)	
Vegetables	58 (43.3%)	173 (47.5%)	
**Physical activity, N (%)**			0.073
Low	47 (35.1%)	121 (33.2%)	
Moderate	51 (38.1%)	108 (29.7%)	
High	36 (26.9%)	135 (37.1%)	
Waist (cm)	95.5 ± 7.0	85.5 ± 8.2	0.000
HC (cm)	100.7 ± 10.6	95.9 ± 6.2	0.000
Weight (kg)	77.4 ± 10.9	65.5 ± 10.6	0.000
BMI (kg/m^2^)	27.1 (22.6~33.7)	24.0 (18.9~31.6)	0.000
SFA (cm^2^)	181.0 ± 61.6	155.5 ± 62.7	0.000
VFA (cm^2^)	111.0 (43.4~214.5)	70.8 (14.2~166.7)	0.000
Fat Mass (kg)	21.7 (15.2~41.9)	16.5 (7.9~34.6)	0.000
SBP (mm Hg)	136 (100~169)	118 (90~150)	0.000
DBP (mm Hg)	85 (67~106)	76 (58~100)	0.000
HR (/min)	76 (60~99)	72 (60~96)	0.002
FPG (mmol/L)	6.0 (4.8~12.1)	5.3 (4.5~10.9)	0.000
0.5-h PG (mmol/L)	10.9 (7.0~20.3)	9.0 (5.5~16.8)	0.000
2-h PG (mmol/L)	8.7 (3.3~21.5)	6.7 (3.8~20.7)	0.000
HbA1c (%)	6.1 (5.1~9.8)	5.8 (4.9~8.8)	0.000
TC (mmol/L)	5.3 (3.3~8.5)	5.0 (3.4~7.7)	0.001
TGs (mmol/L)	2.2 (0.7~13.8)	1.4 (0.6~5.8)	0.000
HDL-C (mmol/L)	1.2 (0.8~1.7)	1.4 (0.8~2.2)	0.000
LDL-C (mmol/L	3.3 (1.3~5.4)	3.1 (1.8~5.0)	0.054
BUA (μmol/L)	364.2 ± 91.5	296.8 ± 88.0	0.000

Normally distributed values are presented as the mean ± SD; skewed values are presented as the median (Q1-Q3).

Alcohol consumption: never, never drink alcohol; occasional, drink less than three times a week; frequent, drink at least three times a week. Diet: meat, meat-based diet; meat/vegetable, meat and vegetarian diet; vegetables, vegetarian diet. Physical activity: low, low physical activity; moderate, moderate physical activity; High, high physical activity.

0.5-h PG, plasma glucose 0.5 hours after a glucose load; 2-h PG, plasma glucose 2 hours after a glucose load; BMI, body mass index; WC, waist circumference; BUA, blood uric acid; FPG, fasting plasma glucose; HC, hip circumference; HR, heart rate; LDL/HDL-C, low-density/high-density lipoprotein cholesterol; SBP/DBP, systolic/diastolic blood pressure; SFA, subcutaneous fat area; TC, total cholesterol; TGs, triglycerides; VFA, visceral fat area.

### Influencing Factors of MetS

**Figure 2:**
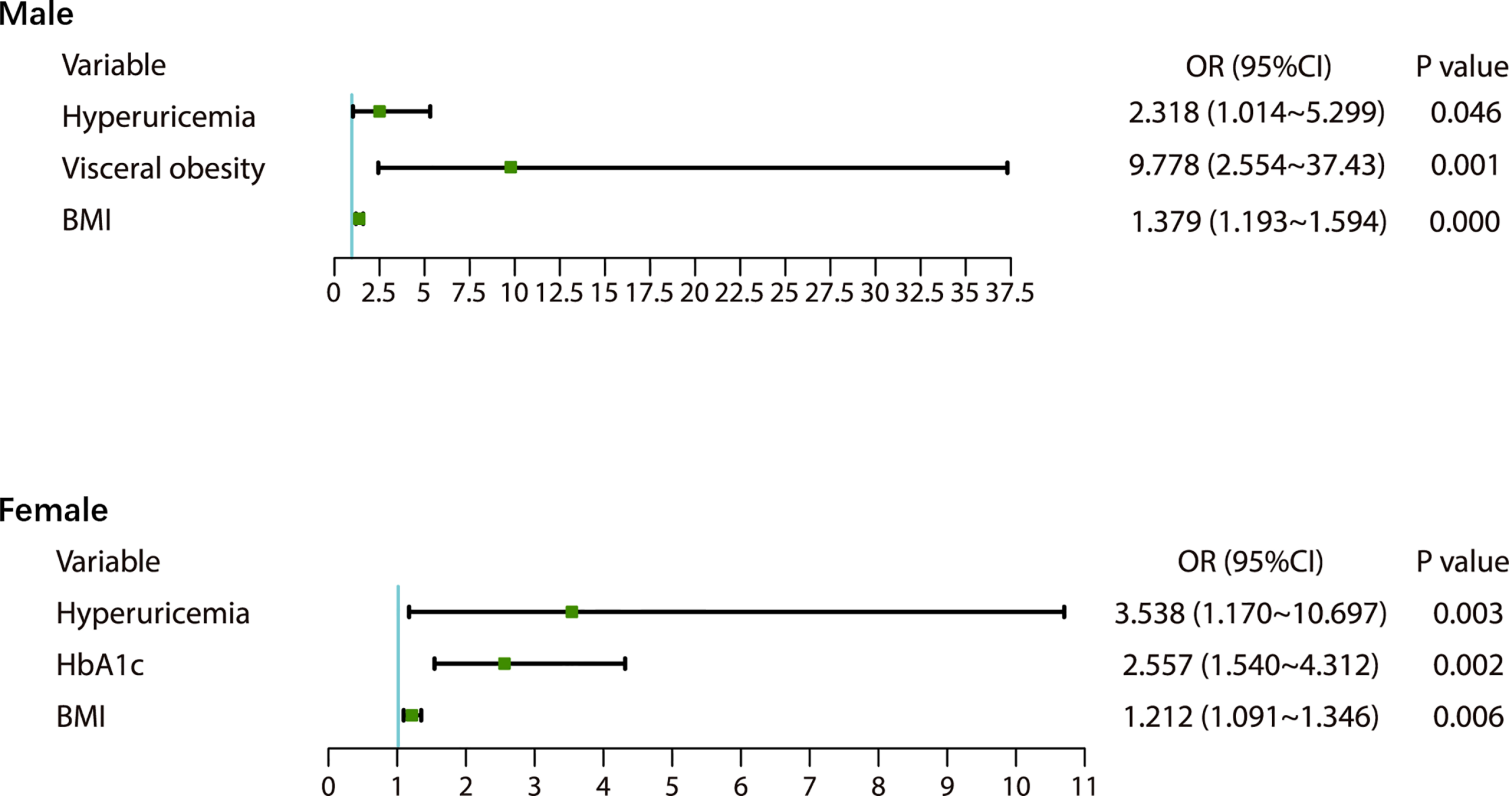
Factors influencing metabolic syndrome in males and females.

There were 98 new cases of MetS during the follow-up period. The cumulative incidence rate of MetS in the survey population was 25.40%; the incidence rate in males was 30.46% and the incidence in females was 23.68%. There was no significant difference in the incidence between men and women (p>0.05). The initial visit characteristics of the population separately are shown in **Table 2**. Alcohol consumption, diet and physical activity before and after follow-up are shown in **Table 3**. We found a significant increase in low-level physical activity and a significant decrease in moderate-level physical activity in patients with newly MetS (p< 0.05) (**Table 3**). Next, we analyzed risk factors that may have influenced the occurrence of MetS within 1.5 years in men and women separately (**Figure 2**). Through univariate logistic analysis, the independent variables affecting the incidence of MetS in men at the initial visit were identified. Multivariate logistic regression was performed with BUA, BMI and visceral obesity as independent variable. We found that BUA was >416 mmol/L (OR=2.318, 95% CI: 1.014-5.299, p<0.05) and high BMI (OR=1.379, 95% CI: 1.193-1.593, p<0.05) among men at the initial visit were associated with the occurrence of MetS (**Figure 2**). Men with visceral obesity were more likely to develop MetS than those with subcutaneous obesity (OR=9.778, 95% CI: 2.554-37.43, p<0.05) (**Figure 2**).

**Table 2:** Incidence of MetS in the non-MetS population during 1.5 years of follow-up.

	Male (n=174)			Female (n=190)		
	MetS	Non-MetS	p	MetS	Non-MetS	p
Participants	53	121	–	45	145	–
Age	52.6 ± 6.1	53.0 ± 7.1	0.658	52.0 (42.2~104.8)	50.0 (43.0~66.4)	0.390
**Menopause, N (%)**			–			0.164
Yes	–	–		22 (48.9%)	54 (37.2%)	
No	–	–		23 (51.1%)	91 (62.8%)	
**Alcohol consumption, N (%)**			0.878			0.623
Never	15 (28.3%)	30 (24.8%)		41 (91.1%)	126 (86.9%)	
Occasional	17 (32.1%)	42 (34.7%)		3 (6.7%)	10 (6.9%)	
Frequent	21 (39.6%)	49 (40.5%)		1 (2.2%)	9 (6.2%)	
**Diet, N (%)**			0.926			0.331
Meat	6 (11.3%)	12 (9.9%)		1 (2.2%)	10 (6.9%)	
Meat/Vegetables	27 (50.9%)	60 (49.6%)		17 (37.8%)	63 (43.4%)	
Vegetables	20 (37.7%)	49 (40.5%)		27 (60.0%)	72 (49.7%)	
**Physical activity, N (%)**			0.178			0.055
Low	10 (18.9%)	28 (23.1%)		12 (26.7%)	68 (46.9%)	
Moderate	20 (37.7%)	29 (24.0%)		18 (40.0%)	43 (29.7%)	
High	23 (43.4%)	64 (52.9%)		15 (33.3%)	34 (23.4%)	
Waist (cm)	92.3 ± 7.2	86.0 ± 6.7	0.000	87.4 ± 9.4	81.9 ± 7.5	0.000
HC (cm)	99.4 ± 5.6	96.1 ± 4.7	0.000	97.4 ± 7.3	94.0 ± 6.3	0.010
Weight (kg)	75.5 ± 9.9	68.4 ± 7.8	0.000	66.1 ± 10.8	59.1 ± 8.5	0.000
BMI (kg/m^2^)	26.4 ± 4.8	23.8 ± 2.4	0.000	25.7 ± 3.5	23.4 ± 3.1	0.000
SFA (cm^2^)	146.8 ± 45.1	114.9 ± 44.5	0.060	202.6 ± 66.1	174.5 ± 60.6	0.015
VFA (cm^2^)	116.2 ± 41.5	73.4 ± 36.0	0.000	76.5 ± 28.7	57.6 ± 24.7	0.012
Fat Mass (kg)	19.2 ± 7.0	15.6 ± 5.6	0.101	22.4 ± 7.9	17.3 ± 6.0	0.144
SBP (mm Hg)	120 (97~158)	120 (92~155)	0.118	118 (90~197)	110 (90~146)	0.007
DBP (mm Hg)	84 (66~106)	76 (58~100)	0.112	78 (60~108)	70 (56~90)	0.000
HR (/min)	72 (60~91)	72 (60~95)	0.795	73 (60~95)	72 (60~98)	0.410
FPG (mmol/L)	5.3 (4.5~12.9)	5.3 (4.3~12.1)	0.977	5.5 (4.6~12.6)	5.2 (4.5~8.0)	0.000
0.5-h PG (mmol/L)	9.5 (6.0~21.4)	9.2 (5.8~19.4)	0.294	10.3 (6.9~16.8)	8.4 (5.4~13.3)	0.000
2-h PG (mmol/L)	7.0 (3.5~25.3)	6.4 (3.2~24.5)	0.178	9.5 (5.3~20.8)	6.6 (4.2~12.7)	0.000
HbA1c (%)	5.7 (4.9~10.8)	5.6 (4.8~9.1)	0.260	6.1 (4.9~9.4)	5.8 (4.9~6.6)	0.002
TC (mmol/L)	5.0 ± 1.0	4.8 ± 0.8	0.134	5.3 (3.7~8.6)	5.0 (3.1~7.7)	0.374
TGs (mmol/L)	1.9 (0.7~12.4)	1.3 (0.6~4.5)	0.000	1.4 (0.5~5.9)	1.2 (0.6~4.9)	0.002
HDL-C (mmol/L)	1.1 (0.7~1.7)	1.3 (0.8~2.1)	0.000	1.4 ± 0.3	1.6 ± 0.4	0.002
LDL-C (mmol/L)	3.3 ± 1.1	3.1 ± 0.7	0.131	3.3 ± 0.8	3.1 ± 0.8	0.132
BUA (μmol/L)	364.0 ± 77.3	334.2 ± 84.6	0.029	280.6 ± 72.3	245.9 ± 65.4	0.005

Normally distributed values are presented as the mean ± SD; skewed values are presented as the median (Q1-Q3).

Alcohol consumption: never, never drink alcohol; occasional, drink less than three times a week; frequent, drink at least three times a week. Diet: meat, meat-based diet; meat/vegetable, meat and vegetarian diet; vegetables, vegetarian diet. Physical activity: low, low physical activity; moderate, moderate physical activity; High, high physical activity.

0.5-h PG, plasma glucose 0.5 hours after a glucose load; 2-h PG, plasma glucose 2 hours after a glucose load; BMI, body mass index; WC, waist circumference; BUA, blood uric acid; FPG, fasting plasma glucose; HC, hip circumference; HR, heart rate; LDL/HDL-C, low-density/high-density lipoprotein cholesterol; SBP/DBP, systolic/diastolic blood pressure; SFA, subcutaneous fat area; TC, total cholesterol; TGs, triglycerides; VFA, visceral fat area.

**Table 3:** Lifestyle change information at baseline and after 1.5 years of follow-up.

	MetS remaining MetS (n=100)			MetS becoming non-MetS(n=34)			Non-MetS becoming MetS(n=98)			Non-MetS remaining non-MetS (n=266)		
	Baseline	Follow-up of 1.5 year	p-value	Baseline	Follow-up of 1.5 year	p-value	Baseline	Follow-up of 1.5 year	p-value	Baseline	Follow-up of 1.5 year	p-value
**Alcohol consumption, N (%)**			0.085			0.505			0.593			0.198
Never	28 (28.0%)	43 (43.0%)		12 (35.3%)	17 (50.0%)		56 (57.1%)	53 (54.1%)		156 (58.6%)	143 (53.8%)	
Occasional	36 (36.0%)	29 (29.0%)		11 (32.4%)	8 (23.5%)		20 (20.4%)	17 (17.3%)		52 (19.5%)	47 (17.7%)	
Frequent	36 (36.0%)	28 (28.0%)		11 (32.4%)	9 (26.5%)		22 (22.4%)	28 (28.6%)		58 (21.8%)	76 (28.6%)	
**Diet, N (%)**			**0.039**			1.000			0.274			0.963
Meat	10 (10.0%)	14 (14.0%)		7 (20.6%)	6 (17.6%)		7 (7.1%)	13 (13.3%)		22 (8.3%)	23 (8.6%)	
Meat/Vegetables	**57^a^ (57.0%)**	**39^a^ (39.0%)**		15 (44.1%)	16 (47.1%)		44 (44.9%)	46 (46.9%)		123 (46.2%)	120 (45.1%)	
Vegetables	**33^a^ (33.0%)**	**47^a^ (47.0%)**		12 (35.3%)	12 (35.3%)		47 (48.0%)	39 (39.8%)		121 (45.5%)	123 (46.2%)	
**Physical activity, N (%)**			0.442			0.610			**0.006**			0.324
Low	24 (24.0%)	46 (46.0%)		13 (38.2%)	11 (8.8%)		**22^a^ (22.4%)**	**40^a^ (40.8%)**		96 (36.1%)	91 (34.2%)	
Moderate	42 (42.0%)	29 (29.0%)		12 (35.3%)	16 (52.9%)		**38^a^ (38.8%)**	**21^a^ (21.4%)**		72 (27.1%)	61 (22.9%)	
High	34 (34.0%)	25 (25.0%)		9 (26.5%)	7 (38.2%)		38 (38.8%)	37 (37.8%)		98 (36.8%)	114 (42.9%)	

The numbers shown in bold are statistically significant, p<0.05.

P-value < 0.05 indicates a significant difference between the groups. ^a^P < 0.05 indicates the pairwise comparison is significant.

Alcohol consumption: never, never drink alcohol; occasional, drink less than three times a week; frequent, drink at least three times a week. Diet: meat, meat-based diet; meat/vegetable, meat and vegetarian diet; vegetables, vegetarian diet. Physical activity: low, low physical activity; moderate, moderate physical activity; High, high physical activity.

Variables affecting the incidence of MetS in women at the initial visit were identified through univariate logistic analysis. Multivariate logistic regression was performed with BUA, BMI and HbA1c as independent variable (**Figure 2**). We found that BUA>356.9 mmol/L (OR=3.538, 95% CI: 1.170-10.697, p<0.05), increased BMI (OR=1.212, 95% CI: 1.091-1.346, p<0.05) and increased HbA1c (OR=2.577, 95% CI: 1.540-4.312, p<0.05) were associated with the occurrence of MetS in women (**Figure 2**).

### Factors Influencing MetS Recovery

**Figure 3:**
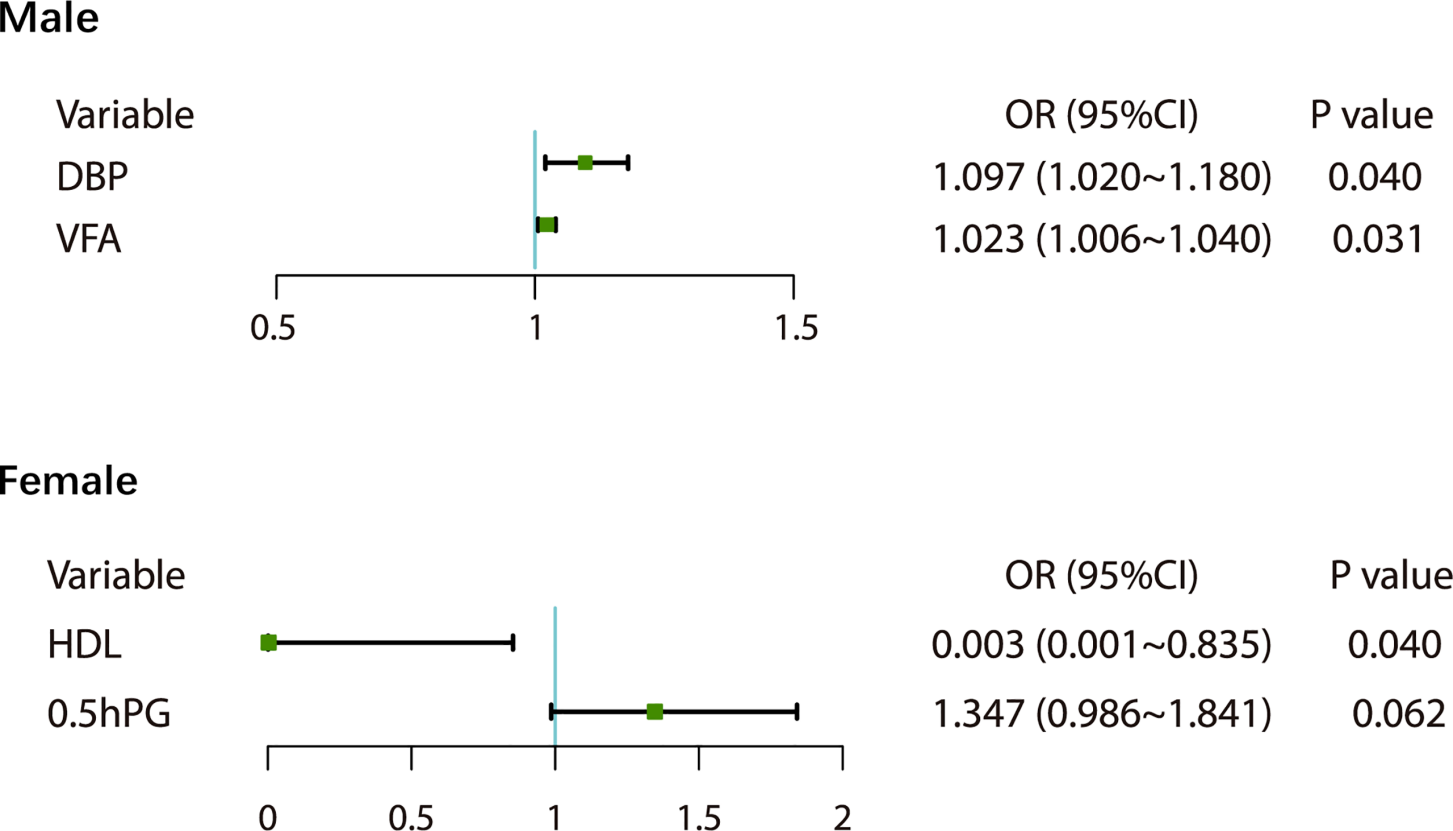
Influencing factors of MetS outcomes in males and females.

At baseline, 204 people with MetS were screened, of whom 134 were followed up, with a follow-up rate of 65.69%. After 1.5 years of follow-up and health guidance and lifestyle intervention, 25.37% of the 134 patients with MetS were classified as non-MetS, including 20 males (21.28%) and 14 females (35.00%). The initial visit characteristics of the population separately are shown in **Table 4**. We found that patients still with MetS after 1.5 years have higher vegetarian habits (p<0.05) (**Table 3**). Through univariate logistic analysis, it was found that the independent variables affecting the outcome of MetS among males were VFA and diastolic blood pressure (DBP), and Multivariate logistic regression was performed with “MetS to non-MetS” as the dependent variable. The results showed that men patients with increased DBP (OR=1.097, 95% CI: 1.020-1.180, p<0.05) and increased VFA (OR=1.023, 95% CI: 1.006-1.040, p<0.05) at the initial visit were unlikely to recover from MetS (**Figure 3**). Through univariate analysis, it was found that the independent variables affecting the outcome of MetS were high-density lipoprotein cholesterol (HDL-C) and OGTT0.5hPG. Multivariate logistic in the female group was performed with “MetS to non-MetS” as the dependent variable. Patients with higher HDL-C at the initial visit were significantly more likely to recover from MetS (β: -3.509, OR=0.003, 95% CI: 0.001-0.835, p<0.05), but elevated OGTT0.5hPG was not associated with the outcome of MetS in women (OR=1.347, 95% CI: 0.986~1.841, p>0.05) (**Figure 3**).

**Table 4:** Outcomes of patients with metabolic syndrome during 1.5 years of follow-up.

	Male (n=94)			Female (n=40)		
	Free MetS	MetS	p	Free MetS	MetS	p
Participants	20	74	–	14	26	–
Age	51.9 ± 5.1	53.2 ± 7.1	0.430	59.1 ± 5.0	57.9 ± 6.1	0.911
**Menopause, N (%)**						1.000
Yes	–	–		8 (57.1%)	14 (53.8%)	
No	–	–		6 (42.9%)	12 (46.2%)	
**Alcohol consumption, N (%)**			0.865			0.404
Never	2 (10.0%)	5 (6.8%)		10 (71.4%)	23 (88.5%)	
Occasional	8 (40.0%)	34 (45.9%)		3 (21.4%)	2 (7.7%)	
Frequent	10 (50.0%)	35 (47.3%)		1 (3.8%)	1 (7.1%)	
**Diet, N (%)**			0.241			0.550
Meat	5 (25.0%)	9 (12.2%)		2 (14.3%)	1 (3.8%)	
Meat/Vegetables	9 (45.0%)	47 (63.5%)		6 (42.9%)	10 (38.5%)	
Vegetables	6 (30.0%)	18 (24.3%)		6 (42.9%)	15 (57.7%)	
**Physical activity, N (%)**						0.519
Low	8 (40.0%)	12 (16.2%)	0.053	5 (35.7%)	12 (46.2%)	
Moderate	5 (25.0%)	34 (45.9%)		7 (50.0%)	8 (30.8%)	
High	7 (35.0%)	28 (37.8%)		2 (14.3%)	6 (23.1%)	
Waist (cm)	96.0 ± 7.8	97.8 ± 5.4	0.220	91.6 ± 9.4	90.9 ± 6.4	0.780
HC (cm)	102 (92~127)	102 (77~112)	0.418	99.2 ± 6.8	97.8 ± 5.7	0.477
Weight (kg)	80.4 ± 10.6	81.8 ± 8.0	0.520	68.3 ± 10.6	67.4 ± 9.1	0.775
BMI (kg/m^2^)	26.5 (22.7~38.0)	27.6 (23.7~33.3)	0.051	26.6 ± 3.6	26.7 ± 3.2	0.926
SFA (cm^2^)	161.3 ± 65.5	167.4 ± 53.7	0.677	220.2 ± 47.3	209.8 ± 68.3	0.619
VFA (cm^2^)	107.1 ± 39.2	131.6 ± 43.5	0.031	91.2 ± 42.7	88.1 ± 34.0	0.806
Fat Mass (kg)	122.8 ± 6.7	122.9 ± 5.0	0.950	23.6 ± 8.5	23.9 ± 7.7	0.500
SBP (mmHg)	133.8 ± 15.1	135.4 ± 14.5	0.662	127.6 ± 18.3	139.5 ± 16.1	0.400
DBP (mmHg)	80 (70~100)	88 (67~110)	0.040	79.0 ± 10.1	86.0 ± 8.3	0.230
HR (/min)	73.8 ± 8.6	76.9 ± 8.3	0.149	74.9 ± 7.3	79.7 ± 12.6	0.198
FPG (mmol/L)	5.7 (4.8~13.6)	6.0 (4.9~12.1)	0.294	5.9 ± 0.9	6.9 ± 2.1	0.089
0.5-h PG (mmol/L)	12.2 ± 3.0	11.5 ± 2.8	0.357	10.0 ± 2.4	12.0 ± 3.1	0.045
2-h PG (mmol/L)	9.1 ± 4.8	9.3 ± 4.2	0.836	9.0 ± 3.1	11.5 ± 5.6	0.133
HbA1c (%)	6.3 ± 1.2	6.2 ± 1.0	0.778	6.1 ± 0.6	6.8 ± 1.0	0.340
TC (mmol/L)	5.2 ± 0.9	5.3 ± 1.2	0.610	5.8 ± 0.9	5.7 ± 1.6	0.789
TGs (mmol/L)	2.0 (0.8~14.2)	2.3 (0.85~14.25)	0.307	1.8 (1.0~4.0)	2.1 (0.6~13.0)	0.244
HDL-C (mmol/L)	1.2 (0.8~1.6)	1.1 (0.8~1.8)	0.754	1.5 ± 0.3	1.3 ± 0.3	0.030
LDL-C (mmol/L	3.0 ± 0.7	3.2 ± 0.9	0.444	3.8 ± 0.7	3.4 ± 1.3	0.251
BUA (μmol/L)	380.2 ± 65.9	400.3 ± 85.1	0.331	285.1 ± 59.4	291.6 ± 72.1	0.776

Normally distributed values are presented as the mean ± SD; skewed values are presented as the median (Q1-Q3).

Alcohol consumption: never, never drink alcohol; occasional, drink less than three times a week; frequent, drink at least three times a week. Diet: meat, meat-based diet; meat/vegetable, meat and vegetarian diet; vegetables, vegetarian diet. Physical activity: low, low physical activity; moderate, moderate physical activity; High, high physical activity.

0.5-h PG, plasma glucose 0.5 hours after a glucose load; 2-h PG, plasma glucose 2 hours after a glucose load; BMI, body mass index; WC, waist circumference; BUA, blood uric acid; FPG, fasting plasma glucose; HC, hip circumference; HR, heart rate; LDL/HDL-C, low-density/high-density lipoprotein cholesterol; SBP/DBP, systolic/diastolic blood pressure; SFA, subcutaneous fat area; TC, total cholesterol; TG, triglyceride; VFA, visceral fat area.

## Discussion

In this prospective cohort study of the Chinese population, we found that within 1.5 years of follow-up, the cumulative incidence of MetS was 30.46% in men and 23.68% in women. Hyperuricemia is a risk factor for MetS in men, and men with visceral obesity are more likely to develop MetS than men with subcutaneous obesity. Women with elevated BMI, hyperuricemia and HbA1c are more likely to develop MetS. Elevated DBP and VFA make it difficult for men with MetS to convert to non-MetS. Elevated HDL-C makes women more likely to recover from MetS.

The association between hyperuricemia and MetS has been explored in many cross-sectional studies, but prospective studies are rare (7–9, 19, 20). Some studies suggest that MetS can increase the incidence of hyperuricemia (19–21); however, others suggest that hyperuricemia should be included as a component of MetS (7). The latest study that reviewed the China Health and Retirement Longitudinal Study (CHARLS) data from 2011 to 2015 concluded that there is a bidirectional relationship between MetS and hyperuricemia, which is in agreement with our research (9). In our study, the OR of female hyperuricemia patients with MetS was greater than that of males, which is in line with the study of American scholar Sui and Turkish scholar Onat et al. that pointed out that the relationship between hyperuricemia and MetS is stronger in females than in males (22). There is currently no direct evidence that lowering BUA levels improves MetS; therefore, larger randomized controlled trials are needed to identify uric acid as a target for MetS prediction and novel therapeutic interventions.

In this study, the incidence of MetS in men with visceral obesity was higher than that in men with subcutaneous obesity. Increased VFA also made men with MetS less likely to recover from MetS. Much evidence supports the hypothesis that visceral fat plays a role in the development of MetS (23–25). In addition, abdominal adipose tissue can secrete inflammatory factors, which are closely related to cardiovascular disease (26). Even the absence of clinical manifestations of obesity can lead to the occurrence of atherosclerosis (27, 28). WC cannot directly determine the content of visceral fat. CT and MRI have limited clinical applications. Therefore, it is necessary to actively use better indicators, such as the visceral adiposity index (VAI) or abdominal volume index (AVI), to improve the identification of visceral obesity (29). More attention should be given to predict the onset and outcome of MetS in men and guide the clinical adoption of more individualized diagnosis and treatment.

Due to the action of estrogen, women generally have higher levels of HDL-C than men (30), and fat is mainly distributed in the buttocks and legs, not the abdomen. During menopause, hormonal changes in women lead to increased visceral fat and elevated blood lipids, which are accompanied by insulin resistance, increased free fatty acid concentrations and increased hepatic lipase activity (31). Epidemiological studies have shown that the prevalence of MetS in older people has tripled in men and increased fivefold in women compared with middle-aged people (32). In a study of the components of MetS, female MetS patients were found to have higher body weight and WC and a higher prevalence of low-density lipoproteinemia (33). This finding is consistent with our results. This shows that the occurrence and prognosis of MetS in women may be driven by high-density lipoprotein. In addition, in this study, no significant differences in the menopause status of women with MetS and non-MetS groups were found, so this needs to be further discussed in future studies.

In this study, 750 people living in Northeast China were followed up for 1.5 years, with a follow-up rate of 66.93%. This prospective research survey with a large sample size can better explain the causal link between influencing factors and diseases. In addition, the age group included in this study was 40-65 years old, which is in the critical period of the body’s transition from middle age to old age. The incidence of MetS was highly variable, and it was more targeted and clinically valuable. In addition, this study is the first to explore the impact of different risk factors on disease outcomes, which is valuable for clinical guidance. This study also included a detailed questionnaire survey, which clearly asked the participants about the status of alcohol consumption, eating habits and physical activity, helping us to rule out confounding factors that might have contributed to the conclusions. In addition, this study surveyed chronic diseases and excluded the influence of medication for diabetes, hypertension hyperlipidemia and hyperuricemia on this study. This study also has certain limitations. The age and geographical limitations of the research subjects affect the generalizability of the research conclusions. The follow-up time of the study was 1.5 years, which could not fully reflect the whole process of the occurrence, development and evolution of MetS. Larger, longer-term prospective studies are needed to investigate the outcome of MetS in the future.

## Data Availability Statement

The raw data supporting the conclusions of this article will be made available by the authors, without undue reservation.

## Ethics Statement

The studies involving human participants were reviewed and approved by Ethics Committee of China Medical University. The patients/participants provided their written informed consent to participate in this study.

## Author Contributions

Conception and design of study: ZS and YL; acquisition of data: YL and HW; analysis and/or interpretation of data: CZ, SF and YL; drafting the manuscript: CZ and SF; revising the manuscript critically for important intellectual content: YL and ZS. All authors contributed to the article and approved the submitted version.

## Funding

This study was funded by the Key Laboratory Project of Thyroid Diseases, National Health Commission (Grant No. 2019PT330001).

## Conflict of Interest

The authors declare that the research was conducted in the absence of any commercial or financial relationships that could be construed as a potential conflict of interest.

## Publisher’s Note

All claims expressed in this article are solely those of the authors and do not necessarily represent those of their affiliated organizations, or those of the publisher, the editors and the reviewers. Any product that may be evaluated in this article, or claim that may be made by its manufacturer, is not guaranteed or endorsed by the publisher.
